# Treatment by ultrasound-guided local infiltration in adhesion-related abdominal pain and intractable hiccups: A case report

**DOI:** 10.1097/MD.0000000000010450

**Published:** 2018-04-20

**Authors:** Dan Zhu, Zhi-Yong Gu, Chia-Shiang Lin, Fa-Chuan Nie, Jian Cui

**Affiliations:** a Department of Pain Care, First affiliated hospital of Third Military Medical University (Army medical University); b Department of Pain Care, Southwest Hospital, Third Military Medical University; c Department of Gastroenterology, The First Affiliated Hospital, Chongqing Medical University, Chongqing, China; d Department of Anesthesiology, Mackay Memorial Hospital, Mackay Medicine, Nursing and Management College, and Mackay Medical College, Taipei, Taiwan.

**Keywords:** hiccups, intra-abdominal adhesion, pain, ultrasound-guided

## Abstract

**Rationale:**

Abdominal pain and hiccups secondary to intra-abdominal adhesion are surgical complications that are often treated by painkillers and secondary surgeries with an unsatisfactory therapeutic effect. This study presents a new treatment method that uses ultrasound-guided local infiltration in peritoneal and abdominal wall adhesions in patients with hiccups and abdominal pain.

**Patient concerns:**

A 62-year-old patient presented to our hospital with a history of intractable hiccups and abdominal pain for 30 years.

**Diagnoses:**

Her abdominal examination revealed a scar with an approximate length of 10 cm on the abdominal umbilical plane; pressing the right scar area could simultaneously induce abdominal pain and hiccups. Intraperitoneal computed tomography examination clearly demonstrated that the bowel had no obvious expansion. Ultrasonographic examination found that peritoneal motility below the normal peritoneal adhesion regions was significantly slower than in the normal regions. The diagnosis of chronic postoperative pain syndrome was clear.

**Interventions:**

The symptoms were significantly alleviated by a successful treatment with ultrasound-guided local infiltration in the peritoneal and abdominal wall scar adhesions.

**Outcomes:**

After 3 stages of hospitalization and 1 year of follow-up, the patient's abdominal wall pain was relieved by approximately 80% and hiccups were relieved by approximately 70%.

**Lessons:**

The above treatment is a useful option for managing abdominal adhesion and accompanying pain or hiccups resulting from abdominal surgery. This method could ease the psychological and economic burden of patients and improve their quality of life.

## Abstract 2

Supplemental Digital Content is available in the text

## 1 Introduction

A high incidence of abnormal adhesions can occur between the parietal and visceral peritoneum, and between various visceral peritonea after abdominal surgery.^[1,2]^ Abdominal pain and hiccups resulting from intra-abdominal adhesions are surgical complications that are often treated by painkillers and secondary surgeries with an unsatisfactory therapeutic effect.^[3]^ This study reports a novel method that uses an ultrasound-guided transversus abdominis plane blockade for treating intra-abdominal adhesion-related pain and hiccups. The study aims to provide a reference for their clinical treatment.

## 2 Case report

**Figure 1:**
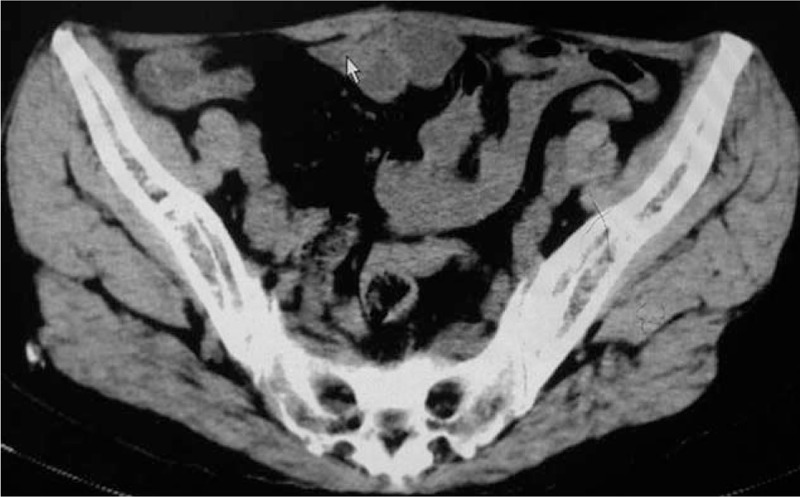
Abdominal CT showed no obvious expansion of the bowel. CT = computed tomography.

A 62-year-old woman was admitted to our department in September, 2015. Thirty years ago, as a result of the national family planning policy, that is, the 1-child policy, she underwent bilateral tubal ligation, but soon experienced irregular pain in the lower abdominal incision area. To alleviate this abdominal pain, she received 3 intestinal adhesion lysis surgeries in a local hospital. Significant pain relief was observed approximately 2 months after the first lysis surgery. However, her abdominal pain became more severe with the gradual occurrence of hiccups after the second intestinal adhesion lysis surgery. Hence, she came to our hospital for help with severe abdominal pain in the right abdominal scar area and unbearable hiccups, and her visual analog scale (VAS) was 6.8 points. Abdominal examination revealed a scar with an approximate length of 10 cm on the abdominal umbilical plane; pressing the right scar area could simultaneously induce abdominal pain and hiccups. Intraperitoneal computed tomography (CT) examination clearly demonstrated that the bowel had no obvious expansion, excluding the possibility of intestinal obstruction (Fig. 1).

After analyzing the symptoms, signs, and characteristics of the pain, we presumed that: the patient's pain area was found at the right abdominal scar area that primarily presented as somatic neuralgia (ie, abdominal wall pain); physical examination showed that pressing the right abdominal wall pain area could induce hiccups, so we temporarily considered the hiccups to be associated with the abdominal wall pain; the nature of the patient's pain was considered to be neuropathic pain complicated by psychogenic pain; the diagnosis of chronic postoperative pain syndrome was clear. Drug treatments including pain relief, sedation, and hiccup relief were used (gabapentin capsules 0.4 g, oral 3 /d; aminophenol oxycodone 1 tablet, oral 2/d; Alprazolam 0.4 mg, oral before bedtime; promethazine hydrochloride injection, 50 mg, intravenous infusion, 1/d). However, with the above treatments, the patient's pain and hiccups did not significantly improve.

We then subsequently applied local infiltration and lysis treatment in the abdominal wall scar combined with a phrenic nerve block under ultrasound guidance. Forty milliliters of anti-inflammatory and local anesthetic drugs (10 mg of triamcinolone acetonide [Jinyao, H20065207], 5 mL of 2% lidocaine hydrochloride [Taiji, H50020038], 5 mL of 0.75% bupivacaine hydrochloride [Hefeng, H31022839], and 30 mL normal saline [Otsuka]) were injected to release the abdominal wall scar. After treatment, her pain was relieved by approximately 50% with a VAS of 2.5 points, but no significant alleviation of the hiccups was found. The treatment outcome showed a strange phenomenon. We initially believed that the hiccups were associated with abdominal wall pain; hence, blocking the pain would, in theory, also inhibit their occurrence. However, the hiccups persisted after releasing the abdominal wall scar within. There could thus be other direct causes of hiccup induction.

We performed ultrasonographic examination and found that peritoneal motility below the normal peritoneal adhesion regions was significantly slower than in the normal regions (Video 1). We further concluded that the patient's hiccups were primarily due to slowed peristalsis caused by peritoneal adhesions. Therefore, the release of the peritoneal adhesions under ultrasound guidance was arranged to relieve the patient's hiccups. This study method was approved by the medical ethics committee of the Chongqing Southwest Hospital. Consent for publication of the clinical details, images, and videos were obtained from the patient. The ultrasound dynamic image (Video 2) shows the puncture needle piercing the patient's peritoneal wall layer within the ultrasound plane, and also 40 mL anti-inflammatory and local anesthetic drugs (the same dose as before) being injected to release the patient's peritoneal adhesion to relieve the patient's hiccups. The patient was advised to visit our hospital for 3 months 1 time, or when the pain and hiccups could not be tolerated after the first discharge. A total of 10 injections and the same treatment of ultrasound-guided local infiltration in the peritoneal and abdominal wall adhesion's scar were given for each stage of hospitalization. After the last time of 3 stages, hospitalization, and 1-year follow-up, the patient's abdominal wall pain was relieved by approximately 80%, hiccups were relieved by approximately 70%, and her VAS had decreased to 1.5 points. We re-performed ultrasound dynamic examination, and found that intestinal peristalsis under the peritoneal adhesion region was faster than before the treatment (Video 3).

## 3 Discussion

To control excessive population growth, China began to implement the family planning policy in 1971; after this, hundreds of millions of reproductive-age women and men were forced to receive fallopian tube or sperm duct ligation surgery, and postoperative abdominal pain occurred in a large proportion of these patients. Furthermore, some patients had decades of abdominal pain with refractory hiccups, as in the case we present here.

The peritoneum primarily consists of mesothelial cells, which are associated with coagulation, fibrinolysis, and the synthesis and degradation of the extracellular matrix; they host defense capabilities as well. Under physiological conditions, fibrinogen and fiber protease inhibitors are equally released by mesothelial cells.^[4,5]^ However, under pathological conditions, such as mechanical injury, tissue ischemia, extrinsic oppression and peritonitis, an increase in inflammatory cells and fibro-serous effusion/exudation leads to fibrin deposition and abdominal adhesion, thereby ultimately inducing abdominal pain or hiccups.^[6,7]^ We analyzed the possible reasons for the curative effects as follows. Our injected steroids have an anti-inflammatory effect in the early stage and can also inhibit the hyperplasia of collagen and granulation tissue in the later stage, diminishing or preventing adhesion and scarring. Injecting a large amount of liquid can release adhesive abdominal scar muscles, reducing abdominal adhesion and further reducing pain. This method of operation does not easily cause secondary damage, as the puncture is performed under visualization. The injection is precisely positioned under ultrasound guidance.

This study presents a patient with abdominal pain and hiccups resulting from abdominal adhesions and slow bowel movements who experienced complete relief after receiving lysis of peritoneal and abdominal wall muscle adhesions under ultrasound guidance. Notably, limitations of the method were found during treatment. A large number of samples are needed to verify their success rate. We used the same method of treatment with great results in a similar case in which the patient experienced 3 months of scar pain and hiccups after pancreatic cancer surgery. Another patient who also had persistent abdominal scar pain after undergoing laparoscopic ligation resulting from ectopic pregnancy 3 years ago was also successfully treated with the same method. Additionally, steroids have anti-inflammatory effects but also an immunosuppressive effect, and appropriate doses in cancer patients remain to be explored.

To our knowledge, this is the first description of this treatment, which may be a useful option in managing abdominal adhesion and accompanying pain or hiccups. This method could ease the psychological and economic burden of patients and improve their quality of life.

## Author contributions

**Conceptualization:** Fa-chuan Nie.

**Data curation:** Dan Zhu, Zhi-yong Gu, Jian Cui.

**Formal analysis:** Zhi-yong Gu.

**Investigation:** Dan Zhu, Zhi-yong Gu.

**Methodology:** Chia shiang Lin.

**Project administration:** Chia shiang Lin, Fa-chuan Nie, Jian Cui.

**Writing – original draft:** Dan Zhu, Zhi-yong Gu.

**Writing – review & editing:** Fa-chuan Nie, Jian Cui.

## Supplementary Material

Supplemental Digital Content

## Supplementary Material

Supplemental Digital Content

## Supplementary Material

Supplemental Digital Content
